# Evaluating the Safety and Effectiveness of Perispinal Etanercept for Post-Stroke Recovery: A Systematic Review

**DOI:** 10.7759/cureus.96707

**Published:** 2025-11-12

**Authors:** Meha Sanghi, Nawat Khanijoun, Nahla Hamza

**Affiliations:** 1 Internal Medicine, Manchester University NHS Foundation Trust, Manchester, GBR; 2 Stroke Medicine, Trafford General Hospital, Manchester, GBR

**Keywords:** cerebrovascular accident, chronic stroke, etanercept, neuroinflammation, perispinal etanercept, post-stroke recovery, stroke, tnf alpha inhibition, tumor necrosis factor

## Abstract

Stroke remains a leading cause of long-term disability in adults, with many survivors experiencing persistent neurological and functional deficits. Conventional rehabilitation offers limited benefit once recovery plateaus. Biological therapies targeting chronic mechanisms of neural dysfunction, such as perispinal etanercept (PSE), a tumor necrosis factor-alpha (TNF-α) inhibitor, have been proposed as potential alternatives. This systematic review evaluates the safety and effectiveness of PSE for neurological recovery in adults with chronic post-stroke deficits. A comprehensive search of PubMed, Embase, MEDLINE, Cochrane Library, Scopus, and Web of Science identified human studies assessing PSE in stroke ≥six months post-event. Two reviewers independently screened, extracted data, and assessed bias using the risk of bias 2 (RoB 2), Newcastle-Ottawa Scale, and Joanna Briggs Institute (JBI) tools. Eligible studies included randomised trials, non-randomised trials, and case-based reports describing neurological, functional, cognitive, or safety outcomes. Five studies met the inclusion criteria: two randomised controlled trials, one large observational cohort, one case series, and one case report. The available evidence showed variable outcomes. Some studies reported short-term improvements in pain and motor function, whereas others found no significant overall functional or quality-of-life gains. Reported benefits, when observed, were typically domain-specific and not consistently sustained. Treatment was well tolerated, with no major safety concerns reported. Robust, large-scale trials are still needed to establish consistent functional benefits and long-term efficacy. Future research should focus on optimising treatment protocols and identifying responsive patient phenotypes through neuroimaging and inflammatory biomarkers. Well-designed multicenter trials with standardised stroke-specific endpoints and extended follow-up are essential to determine the true therapeutic value of PSE before routine clinical adoption.

## Introduction and background

Growing evidence indicates that the brain retains the capacity for functional reorganisation and repair well beyond the acute phase of stroke. As a result, chronic stroke is increasingly understood not as a static state of irreversible damage but as an active and potentially modifiable phase of neurological dysfunction. During this stage, many patients continue to experience persistent deficits, including spasticity, motor weakness, central post-stroke pain, and cognitive decline, which contribute to long-term disability. Despite major advances in acute stroke treatment, few effective therapies exist for improving outcomes in the chronic phase, underscoring the urgent need for interventions [1-3]. 

Tumour necrosis factor-alpha (TNF-α), a key inflammatory cytokine, rises sharply after ischaemic injury within peri-infarct and remote cortical regions due to microglial activation, thereby disrupting the blood-brain barrier integrity and sustaining a cycle of neuroinflammation [4]. Persistent TNF-α signalling disrupts synaptic function and impairs cortical reorganisation, mechanisms thought to underlie chronic neurological deficits and central post-stroke pain [5]. Etanercept, a recombinant TNF-α inhibitor approved for systemic inflammatory diseases [6], has therefore been proposed as a potential therapy to modulate these processes, thereby restoring a more adaptive neuroimmune environment and supporting neural recovery [5].

Because etanercept does not cross the blood-brain barrier efficiently, perispinal administration has been investigated as a means of indirect central delivery. The technique involves injecting the drug into the soft tissues around the spine, where venous connections to the cerebrospinal system may permit access to the brain [7,8]. 

Preliminary clinical reports, ranging from individual case studies to larger observational cohorts, have described rapid improvements in motor function, spasticity, cognition, and pain following perispinal etanercept (PSE), even when administered years after the stroke [9-11]. These findings have generated considerable interest among patients and clinicians, but the approach remains investigational. To date, regulatory authorities have not approved etanercept for post-stroke neurological recovery, and professionals have emphasised the need for rigorous controlled trials to validate its efficacy [12]. This has prompted the initiation of randomised controlled trials (RCTs) designed to definitively assess its therapeutic value [13,14]. In light of the growing yet heterogeneous evidence base, we undertook a systematic review to critically evaluate current evidence on the safety and efficacy of PSE after stroke and identify knowledge gaps to guide future research. 

## Review

Methods 

Search Strategy 

A comprehensive literature search was performed on PubMed, Embase, MEDLINE, Cochrane Library, Scopus, and Web of Science databases through September 2025 using the keywords: “stroke” AND “etanercept” AND “perispinal” with no restrictions on publication date or language. The search strategies incorporated controlled vocabulary and subject headings appropriate to each database (e.g., Medical Subject Headings (MeSH) in PubMed and MEDLINE, Emtree terms in Embase) combined with free-text terms to maximise retrieval sensitivity. Only human studies were included. Grey literature sources were also searched, and unpublished or ongoing studies were identified through clinical trial registries. Conference abstracts were screened, but were excluded during full-text eligibility assessment due to insufficient methodological and outcome detail for appraisal. The review protocol was registered but not peer-reviewed on the International Prospective Register of Systematic Reviews (PROSPERO; ID: CRD420251171901). The Preferred Reporting Items for Systematic Reviews and Meta-Analyses (PRISMA) flow diagram was utilised to outline the process (identification, screening, eligibility, and inclusion) for selecting relevant studies [15]. 

Data Extraction 

Two independent reviewers assessed the eligibility of each study by screening the titles and abstracts. Full-text reviews of selected studies were performed, and data were extracted using a standardised data extraction form. Because the eligibility criteria were narrowly defined, only minimal disagreement occurred during study selection, and no adjudication was required; therefore, formal inter-rater reliability was not calculated. Any discrepancies were resolved by discussion until consensus was reached. 

*Eligibility Criteria and PICO (Population, Intervention, Comparison, Outcome) Framework* 

The review was guided by a clearly defined PICO framework. The population consisted of adults (≥18 years) with a history of ischaemic or haemorrhagic stroke beyond the acute phase (≥six months post‑stroke) and had persistent neurological or cognitive deficits. The intervention was perispinal administration of etanercept alone or in combination with standard rehabilitation. The comparison included placebo, standard care, or no treatment. Eligible studies reported at least one relevant outcome, such as neurological improvement (e.g., Fatigue Assessment Scale (FAS) or modified Rankin Scale (mRS)), functional recovery, cognitive outcomes (e.g., Montreal Cognitive Assessment (MoCA)), quality of life, pain, spasticity, mood, or safety outcomes. Original clinical studies such as randomized or non‑randomised trials, cohort studies, case series, and case reports were included.

The exclusion criteria were as follows: (1) studies that did not meet the above inclusion criteria, including those involving paediatric or acute post‑stroke populations, animal models, other routes of etanercept administration or other anti‑TNF-α agents; (2) study designs that were not original clinical studies, such as systematic reviews, meta‑analyses, narrative reviews, editorials, or study protocols without patient‑level data; and (3) studies lacking sufficient data on neurological, functional, cognitive or safety outcomes. 

Risk of Bias Assessment 

To evaluate the methodological quality of the included studies, two reviewers independently applied established critical‑appraisal tools appropriate to each study design. The Cochrane risk of bias (RoB) 2 tool [16] was used for randomised trials. The Newcastle-Ottawa Scale (NOS) [17] was used to evaluate non‑randomised controlled or observational studies. Joanna Briggs Institute's (JBI) critical appraisal checklist [18] was used for case series and case reports. Each tool generated a score across specific domains (e.g., reporting, external validity, internal validity), and studies were classified as high, moderate, or low quality based on these scores. For the NOS, scores of ≥seven were considered low risk, five to six were moderate, and ≤four was high risk. For the RoB 2 and JBI tools, overall judgments were derived from the domain ratings: a study was rated “low risk” only if all domains were low risk, “some concerns/moderate” if one or more domains showed minor issues, and “high risk” if any domain indicated a serious methodological flaw. Any discrepancies between reviewers were resolved through discussion or consultation with a third reviewer. To minimise publication bias, we additionally searched trial registries including ClinicalTrials.gov, the EU Clinical Trials Register (EUCTR), and the WHO International Clinical Trials Registry Platform (ICTRP) for unpublished or ongoing studies. 

Results 

Search Results 

**Figure 1:**
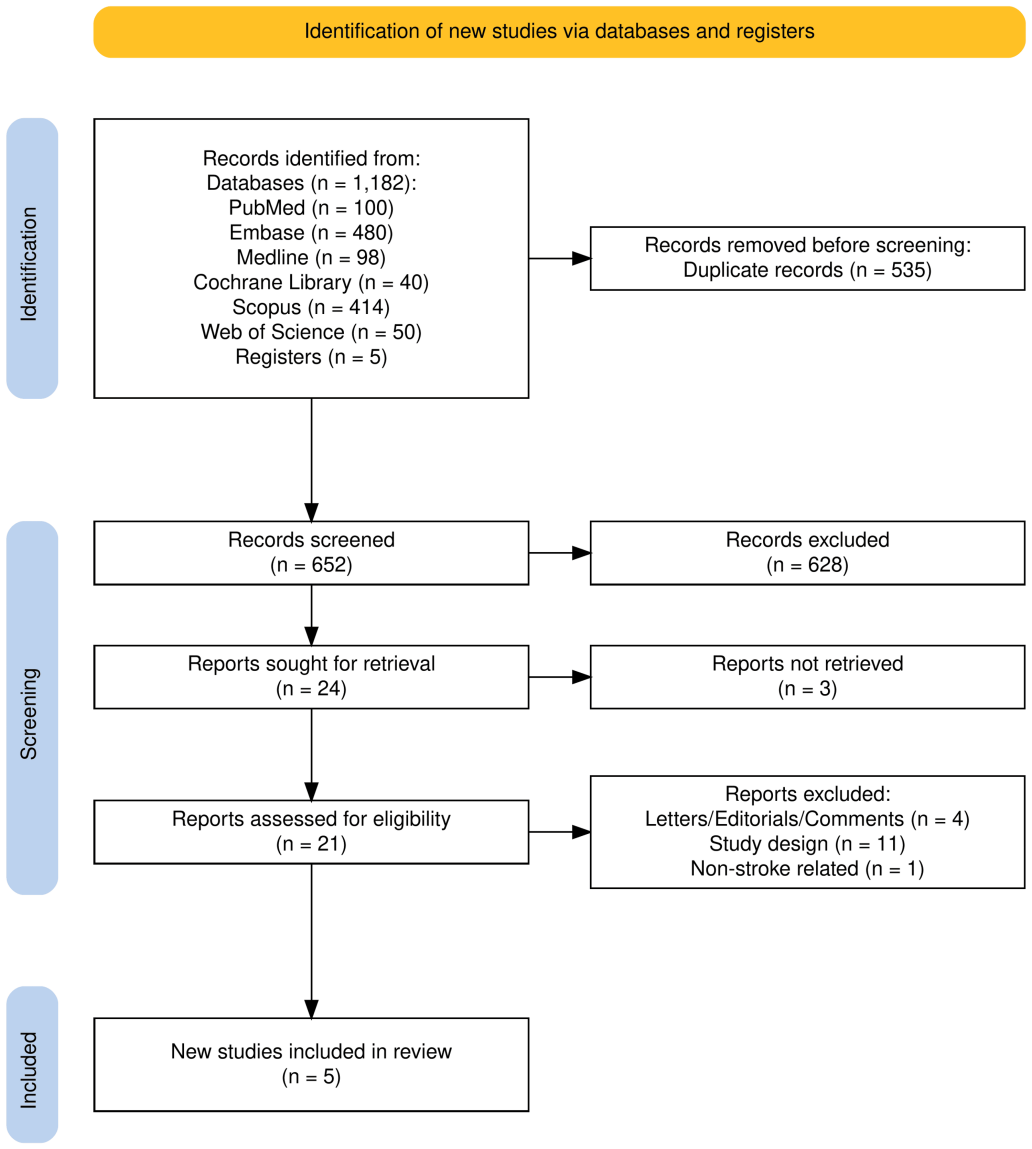
PRISMA flowchart showing study selection process PRISMA: Preferred Reporting Items for Systematic Reviews and Meta-Analyses.

We screened 1,182 records from PubMed (100), Embase (480), MEDLINE (98), Cochrane Library (40), Scopus (414), and Web of Science (50). Searches of trial registries yielded five records. After removal of duplicates, 652 records remained. A total of 628 records were excluded based on titles and abstracts, leaving 24 for which full texts were sought. We obtained the full text of 21 records, while three records could not be retrieved. Upon further analysis of full texts, we excluded 16 studies based on the pre-specified criteria, and five studies were incorporated in our final review (Figure 1). 

Study Characteristics 

The study characteristics are summarised in Table 1.

**Table 1:** Characteristics of the included studies Summary of study design, population, intervention, efficacy, and safety outcomes for all studies evaluating perispinal etanercept in chronic post-stroke patients; TBI: Traumatic brain injury; RCTs: Randomised controlled trials; AE: Adverse event; FAS: Fatigue Assessment Scale; MoCA: Montreal Cognitive Assessment; VAS: Visual analogue scale; MCA: Middle cerebral artery; AESI: Adverse effect of special interest; QoL: Quality of life; SF-36: Short form 36.

Author & Year	Study design/Study size	Population	Treatment regimen	Efficacy	Safety outcomes	Other key outcomes
Tobinick (2011) [10]	Case series/N=3	Adults (mean age 63 y) with chronic post-stroke deficits (13–36 months post-stroke)	Perispinal etanercept 25 mg – single dose in 1 patient, 2 doses in 2 patients (~3 weeks apart)	All 3 (100%) showed rapid motor, speech, and cognitive improvement within 10 min after the first dose; benefits were maintained for ≥1 month	No adverse events reported (0%)	Qualitative improvements in gait, limb strength, and behaviour; uncontrolled observations
Tobinick et al. (2012) [9]	Observational/N=629 (617 stroke, 12 TBI)	Mean age 65.8 y; mean time since stroke 42 months (range 0.5–419); ≈31 % <6 months post-stroke	Single or repeated perispinal etanercept 25 mg	Domain-specific proportions of patients showing improvement ranged approximately from 50% (pain) to >90 % (motor function); p<0.001 (FAS, MoCA, 20 m walk)	Adverse events not systematically reported (<1% injection discomfort noted)	Largest dataset; uncontrolled; mean follow-up ≈3 weeks; included 1 participant <18 y and ≈31 % <6 months post-stroke (retained due to negligible impact on overall results)
Ralph et al. (2020) [14]	Phase I/II Double-blind RCT /N=22 (10 etanercept, 12 placebo)	Adults (30–80 y) with chronic post-stroke pain (≈4–5 years after stroke)	Perispinal etanercept 25 mg × 2 (Day 1 & Day 14) vs saline placebo	Pain VAS ↓19.5–24 points (24–30% reduction, p<0.05); shoulder flexion ↑55° after 2 doses (p=0.003)	No serious AEs (0%); one AESI (shingles), classified as possible treatment-emergent risk → participant withdrawn	Short-term benefit in pain and mobility; effects sustained to Day 30
Tobinick (2020) [11]	Case report/N=1	59-y male; right MCA stroke 16 y prior	Single perispinal etanercept 25 mg	Complete resolution of pain (10 min) and neglect (30 min); FAS increased by 29% (24→31) at 2 months; sustained benefit 11 months.	None reported	Improved gait, spasticity, attention; relief sustained 11 months
Thijs et al. (2025) [13]	Multicenter Double-blind RCT/N = 126 (63 etanercept, 63 placebo)	Ischaemic/haemorrhagic stroke 1–15 years post; median age 54.5 y	Perispinal etanercept 25 mg (posterior cervical C6–C7/C7–T1); randomised to receive either 1 dose or 2 doses 28 days apart (placebo-matched schedule); primary assessment at day 28 after first injection; follow-up to day 56.	QoL response (≥5-point SF-36 improvement): 53% vs 58% (OR 0.82, 95% CI 0.40–1.67, p=0.58) → no efficacy signal	Serious AEs similar (etanercept 2%, placebo 3%); no new safety concerns	Class I evidence of no benefit on QoL; short-term safety confirmed

Of the five included studies, there were two RCTs, one large observational cohort, one case series of three patients, and one case report. The studies were conducted in Australia, New Zealand, and the United States. Across all studies, the weighted average of reported mean or median ages was approximately 64 years (range 30-97 years) [9-11,13,14]. One study reported a single participant younger than 18 years; given the negligible proportion and minimal impact on overall age distribution, this case was retained for completeness [9]. The majority of patients had ischaemic strokes, accounting for approximately 60.4% of all participants across the included studies, while 27.2% presented with haemorrhagic strokes and 12.4% with unspecified aetiologies. The time since stroke onset varied widely, from as early as six months to more than 15 years post-event, with most studies focusing on individuals at least one year after stroke in whom neurological deficits had stabilized. The observational cohort study included 31% of participants less than six months post-stroke, but as the observational dataset did not report outcomes stratified by chronicity, it was retained for completeness. The data were interpreted with caution, given partial overlap with the review’s inclusion criteria. 

All studies used etanercept 25 mg administered via a standardized perispinal injection technique. Administration was performed into the posterior cervical interspinous space (typically between C6-C7 or C7-T1) with the patient positioned briefly (four to five minutes) in a Trendelenburg posture to enhance cerebrospinal venous flow toward the brain. The dosing schedule varied: a single 25 mg injection was used in the case report [11], two injections 14 days apart in the Phase I/II trial [14], and individualised or repeated dosing (often one or two injections several weeks apart) in the case series and observational cohort [9,10]. The multicentre RCT compared single- versus double-dose (day 0 and day 28) regimens [13]. 

Risk of bias in the Included Studies 

The findings of risk of bias assessment are summarised in Tables 2, 3.

**Table 2:** Risk of bias assessment for randomised controlled trials (RCTs) and observational studies

Author & Year	Study design	Tool used	Randomisation/Allocation	Blinding/Deviations	Missing outcomes	Outcome measurement/Reporting	Other domains	Overall risk of bias
Thijs et al. (2025) [13]	Multicenter RCT	Cochrane RoB 2	Low	Low	Low	Low	—	Low risk
Ralph et al. (2020) [14]	Phase I/II RCT	Cochrane RoB 2	Some concerns	Low	Low	Some concerns	—	Some concerns
Tobinick et al. (2012) [9]	Observational cohort	Newcastle–Ottawa Scale (NOS)	Few	Few	Few	Few	Short follow-up period	High risk

**Table 3:** Risk of bias assessment for the case series and case reports JBI: Joanna Briggs Institute.

Author & Year	Study design	Tool used	Selection/Inclusion criteria	Reporting of clinical details	Outcome reporting/Follow-up	Confounding/Validity	Overall Risk of Bias
Tobinick (2011) [10]	Case series	JBI checklist for case series	Partial	Yes	Limited	Unclear	High risk
Tobinick (2020) [11]	Case report	JBI checklist for case reports	N/A	Yes	Yes	Unclear	High risk

The Perispinal Etanercept for Stroke Outcomes Study (PESTO; Thijs et al., 2025) [13] was rated low risk across all domains, whereas the Phase I/II RCT (Ralph et al., 2020) [14] showed some concerns for risk of bias due to its small sample size, early termination, and reliance on several non-validated or assessor-dependent secondary outcome measures, although randomisation and blinding were otherwise adequate. The large observational cohort by Tobinick et al. (2012) [9] was rated as high risk of bias due to its uncontrolled, single-centre design, lack of blinding, self-reported outcomes, and short follow-up period, which limited internal validity despite the large sample size. The case series by Tobinick (2011) [10] was assessed as high risk of bias due to unclear inclusion criteria, potential selection bias, and reliance on subjective, unblinded outcome reporting with limited follow-up, despite detailed clinical descriptions. The single-patient case report by Tobinick (2020) [11] was also rated high risk of bias due to its uncontrolled design, potential conflict of interest, and lack of independent outcome verification, although it provided comprehensive clinical details and adequate follow-up duration.

Motor Function and Quality of Life Outcomes

Across the included studies, improvements in motor recovery following PSE were reported with varying magnitude. In the 2011 case series [10], all three participants demonstrated enhanced coordination and gait within minutes of injection, persisting for several weeks. The study by Tobinick et al. (2012; n=617) [9] documented immediate improvement in the 20-m walk time of the stroke cohort by a mean reduction of 7.5 seconds (median -3.2; p<0.0001) from baseline, persisting at one and three weeks, along with significant gains in the mRS and Activities of Daily Living (ADL) scores (both p<0.001), while emotional responsiveness and engagement were reported to have subjectively improved. The Phase I/II RCT (Ralph et al., 2020; n=22) [14] reported significant gains in paretic-arm shoulder flexion (~30° after the first dose, p=0.003; ~55° after the second dose, p=0.001) and modest improvements in mood-related and behavioural measures. The PESTO Study (Thijs et al., 2025) [13] evaluated health-related quality of life using the 36-Item Short Form Health Survey (SF-36v2) and found no significant between-group difference in the proportion of participants achieving a ≥five-point improvement at 28 days (etanercept 53% vs placebo 58%; adjusted OR 0.82, 95% CI 0.40-1.67; p=0.58). The mental health component of the score also showed no significant change. 

Pain Outcomes 

Analgesic effects were among the most consistent findings. In the Phase I/II RCT (Ralph et al., 2020) [14], PSE produced significant reductions in average and worst pain by day 30 (approximately 15 points versus placebo, p=0.023-0.04), with an immediate median decrease of ~27.5 points within 30-60 minutes of dosing in the active arm. In the single-patient case report (Tobinick, 2019) [11], central post-stroke pain resolved completely within 30 minutes after a single 25 mg injection and remained absent for 11 months. In the large observational cohort (Tobinick et al., 2012; n=617 stroke patients) [9], mean visual analogue scale (VAS) pain scores declined from 7.1 at baseline to 3.1 immediately, 4.0 at one week, and 2.3 at three weeks (all p≤0.0012); the proportion of participants reporting pain improvement increased to 72% at one week and 73% at three weeks (p<0.0001). The PESTO Study (Thijs et al., 2025) [13] did not assess pain as a primary endpoint, though an exploratory analysis showed a modest median reduction in VAS pain scores (−9.5 points at day 56). 

Cognitive Function Outcomes 

The 2011 case series [10] reported improved speech, attention, and executive function. Tobinick et al. (2012) [9] observed significant changes in the MoCA scores with an increase of 2.6 points immediately post-treatment, 2.9 points after one week and 3.5 points after three weeks (p<0.001). The 2020 case report [11] documented rapid resolution of hemispatial neglect on the Clock-Drawing Test. Cognitive outcomes were evaluated in both randomised trials. In the Phase I/II study (Ralph 2020) [14], the MoCA score increased modestly (~+1 point) after etanercept but not significantly compared with placebo. In the PESTO Study (Thijs et al. 2025) [13], median MoCA scores remained unchanged at day 56 in both study groups (p=0.49). 

Safety Outcomes 

PSE was generally well tolerated across all included studies, with no serious treatment-related adverse events reported. Ralph et al. (2020) [14] reported just one adverse event of special interest (AESI), a case of shingles (herpes zoster) was reported after the first dose and was considered a possible treatment-emergent risk. The affected participant was withdrawn from the trial, and no additional systemic or local adverse effects were reported. All other included studies [9-11,13] reported no adverse events following PSE. 

Discussion 

PSE has been explored as a therapy for chronic stroke deficits with mixed results in this review. The latest evidence from the double-blind RCT (2025) found the intervention safe but not superior to placebo for overall post-stroke outcomes [13]. Noteworthy positive signals in other studies were improved motor function and cognition, decreased post-stroke pain, and enhanced post-stroke quality of life [9-11]. These observations suggest that the translation of PSE’s anti-inflammatory mechanism into broad neurological recovery was not fully supported by high-quality trial data, but they do indicate that etanercept’s benefits may emerge in specific domains. 

The early literature, exemplified by Tobinick’s case series and large observational cohort, consistently described rapid and multidomain neurological recovery within minutes of PSE administration, often persisting for weeks to months [9,10]. Although these reports captured compelling clinical narratives, their methodological limitations, including a lack of blinding, subjective outcome measurement, and potential conflicts of interest, limit the strength of causal inference. The magnitude and immediacy of the reported effects raise concerns about expectancy bias and the placebo effect, particularly given the invasive and attention-intensive nature of the intervention, as it requires clinical expertise and vigilant monitoring of the patient.

The first double-blind, placebo-controlled evidence emerged from the Phase I/II RCT conducted by Ralph et al. (2020), which provided a more structured examination of PSE’s effects [14]. Despite a small sample size, the trial demonstrated statistically significant short-term pain reduction and increased shoulder flexion in the active arm compared with placebo. Nonetheless, the trial’s exploratory scope, limited power, and short follow-up constrain the generalisability of its conclusions. The study also did not find significant improvement in global cognitive or mood measures, suggesting that the treatment’s effects, if any, may be domain-specific rather than generalised across neurological function.

In contrast, the recent PESTO trial (Thijs et al., 2025) provided the most rigorous and contemporary evaluation to date [13]. With multicentre conduct, concealed allocation, and blinded outcome assessment, the trial reported no improvement in quality of life 28 days after treatment. Neither the single-dose nor the repeated-dose regimen demonstrated superiority over placebo in SF-36v2 or cognitive endpoints. This null result strongly challenges the reproducibility of earlier findings and underscores the need to ascertain genuine pharmacological effects. Importantly, the PESTO trial was not powered to detect subtle domain-specific changes in pain or spasticity as primary outcomes, except an exploratory analysis that showed reductions in pain score, leaving some mechanistic hypotheses incompletely tested. 

Pain reduction was consistently observed across all studies, including the two RCTs, indicating a potential analgesic effect [9-11,13,14]. Bo et al. (2022) noted that central post-stroke pain (CPSP) remains challenging to manage [19], with limited high-quality evidence supporting current pharmacological options, such as amitriptyline, anticonvulsants, pregabalin, opioids, and corticosteroids, due to little sustained benefit and significant adverse effects [19-23]. In contrast, anti-TNF biologics target neuro-immune inflammation rather than neural transmission, the primary mechanism of action of existing pharmacotherapies [5,9]. TNF inhibitors have also been observed to improve fatigue symptoms in chronic inflammatory disease [6]. These comparisons highlight that the multi-modal immunological action of etanercept could address gaps left by symptom-specific treatments, if future evidence confirms a benefit.

Interpretation of the results should consider marked heterogeneity among study participants. Differences in time since stroke onset (ranging from six months to over 15 years), stroke subtype, and baseline disability likely influenced responsiveness to therapy. Earlier or less-severe cases may exhibit greater neuroplastic potential, whereas the impact in patients many years post-stroke may demonstrate attenuated benefit despite similar mechanistic targets.

Safety outcomes across all studies were reassuring, with no serious treatment-related adverse events reported [9-11,13,14]. The absence of major systemic toxicity aligns with the well-established tolerability of etanercept in other indications. However, the safety evidence base remains limited, as most participants received only one or two doses with short-term follow-up. Longitudinal surveillance and standardised adverse-event reporting are needed before long-term neurorehabilitative use can be considered. Even though etanercept appears acutely safe in these trials [9-11,13,14], scaling up its use would require robust safety data with trials focusing on safety as a primary outcome, particularly in a population potentially vulnerable to serious infections or systemic immune reactions. 

Given the above considerations, a measured but sustained approach is warranted to further explore TNF-α inhibition for stroke recovery. The 2025 randomized controlled trial was terminated early, raising the possibility of insufficient power to detect smaller benefits [13]. Moreover, participants had heterogeneous chronic deficits (one to 15 years post-stroke, varying disability levels). If etanercept’s effects are confined to certain post-stroke complications (e.g., central pain or inflammation-driven symptoms) [24], a trial in an unselected population may dilute those benefits. Indeed, the authors noted the need for more precise patient selection and timing, and future trials can focus on targeted patient selection. The study also had a short follow-up duration, whereas smaller studies suggest repeated dosing and longer observation may reveal cumulative improvements [9-11,14]. Future studies should evaluate multi-dose regimens over a longer follow-up period with rigorous blinding and objective outcome metrics. Neuroimaging, biomarker, and pharmacokinetic endpoints can be incorporated to clarify whether PSE reaches central targets and modulates neuroinflammatory pathways. A trial focusing on CPSP could definitively confirm whether etanercept is efficacious for that indication. Another avenue is to compare etanercept to existing treatments in controlled trials. Similarly, comparing PSE plus rehabilitation versus rehabilitation alone in motor recovery could show any additive benefit of the drug. Thus, while a high-quality trial failed to show broad efficacy, it would be premature to completely discard PSE as a therapy. Instead, these results highlight the importance of refining our approach, identifying which patients and outcomes are most likely to respond to TNF-α inhibition, and confirming any niche benefits with further research.

Strengths and limitations

Our systematic review applied rigorous methodology, including a comprehensive search strategy and inclusion of unpublished data sources, as well as independent screening by two reviewers to reduce selection and publication bias. This review offers a comprehensive synthesis of both controlled and observational data on PSE in chronic stroke, including the most recent multicentre RCT evidence [13]. Additional strengths include a structured risk of bias assessment and critical comparison across diverse study designs. 

Limitations include the wide variation across studies in design, quality, and sample size, with some lacking blinding, standardised outcomes, or adequate follow-up. Evidence for many domains (e.g. fatigue, mood, and function) remains anecdotal or uncontrolled [9,10]. The generalisability of findings is limited by geographic and demographic diversity. Publication bias cannot be ruled out, and many outcome measures were subjective. A pooled or quantitative synthesis was not performed due to differences in outcome measures and assessment time points between studies. As these outcomes were not reported using comparable scales, results were summarised qualitatively at the study level. Furthermore, long-term safety data for PSE in neurological populations remain scarce. 

## Conclusions

PSE has mechanistic plausibility and emerging signals of benefit. However, the overall evidence remains inconclusive, with only one large RCT to date and marked variability across smaller studies. While current data do not support routine clinical use, early analgesic effects and biological rationale provide justification for further exploration. Future large-scale RCTs and biomarker-driven research will be critical in defining the role of PSE in post-stroke care. In the broader context, the concept of immune modulation to enhance neurological recovery remains attractive and is being explored in related conditions. Even if etanercept ultimately proves ineffective for most stroke survivors, these studies will yield valuable insights into the role of inflammation in stroke recovery. On the other hand, if ongoing research identifies a subgroup that benefits, for example, patients with chronic post-stroke neuropathic pain, it could open a novel therapeutic avenue and improve quality of life for those individuals. 
